# Repeatability of nerve conduction measurements derived entirely by computer methods

**DOI:** 10.1186/1475-925X-8-33

**Published:** 2009-11-06

**Authors:** Xuan Kong, Eugene A Lesser, Shai N Gozani

**Affiliations:** 1 NeuroMetrix, Inc 62 Fourth Avenue, Waltham, MA, USA

## Abstract

**Background:**

Nerve conduction studies are an objective, quantitative, and reproducible measure of peripheral nerve function and are widely used in the diagnosis of neuropathies. The purpose of this study is to determine the reliability of nerve conduction parameters derived entirely from computer based data acquisition and waveform cursor assignments and to quantify the relative contributions of test variability sources.

**Methods:**

Thirty volunteers, some with symptoms suggestive of neuropathies; of these, 29 completed the study. The median, ulnar, deep peroneal, posterior tibial, and sural nerves were evaluated bilaterally at two test sessions 3-7 days apart. Within each session, nerves were tested twice within 10 minutes. The analyzed nerve conduction parameters include motor latencies, motor conduction velocity (CV), compound muscle action potential (CMAP) amplitude, F-wave latencies (minimum, mean and maximum), sensory peak latency (DSL), sensory CV, and sensory nerve action potential (SNAP) amplitude. The primary outcome measure is variance component analysis and the corresponding coefficient of variation (CoV). The between-session-test variance is the sum of within-session variance and between-session variance, quantifying the total variation between test sessions. Additional statistical measures include the intraclass correlation coefficient (ICC) and relative interval variation (RIV).

**Results:**

Motor and sensory latencies, CV and F-wave latency parameters have low between-session-test CoVs, ranging from 4.2% to 9.8%. Amplitude parameters have a higher between-session-test CoVs in the range of 15.6--19.8%. Between-test CoVs are about 30--80% lower than between-session CoVs with the exception of F-wave latency parameters. Between-test ICC values are 0.96 or above for all parameters. Between-session ICC ranges from 0.98 for F-wave latency to 0.77 for sural sensory CV. All latency-related between-session ICCs have a value 0.83 or above. The RIVs are the tightest for F-wave latency parameters and widest for CMAP amplitude parameters. Repeatability in a sub-group of subjects with more severe symptom grades follows the same trend as the overall study population without substantial quantitative differences.

**Conclusion:**

The study demonstrates the high repeatability of nerve conduction parameters acquired by modern electrodiagnostic instruments using computer based waveform cursor assignment. The reliability is comparable to benchmark studies in which the nerve conduction measurements were performed manually in controlled multi-center clinical trials. Furthermore, the ranking of reliability, whereby F-wave latencies have the best reproducibility and amplitudes the worst, is also consistent with the benchmark studies.

## Background

Nerve conduction studies (NCS) are an objective, quantitative, and reproducible measure of peripheral nerve function and are widely used in the diagnosis of neuropathies [1]. They have also been used to monitor neuropathic disease progression [2] and the efficacy of interventions in clinical trials [3,4]. Several sources of test variability may degrade NCS measurement repeatability. They include the use of disparate equipment at different test sessions or sites [5], inconsistent placement of recording and stimulating electrodes [6], use of non-standardized distance measurements, differences or errors in waveform cursor assignments, use of sub-maximal electrical stimuli, poor skin preparation resulting in high skin impedance, and failure to either maintain limb temperature within an acceptable range or to compensate for temperature deviations. All these factors may compromise repeatability and lead to inaccurate diagnostic conclusions.

Aside from inherent physiological changes, factors that influence repeatability of NCS measurements are broadly grouped into two categories: inter-tester variability and intra-tester variability. Inter-tester variability refers to variability of a NCS parameter measured on a single individual when repeat test measurements are made by two or more examiners. Intra-tester variability refers to variability of a NCS parameter when repeat test measurements are made by a single examiner. It has been suggested that both types of variability may be minimized with measurement standardization and incorporation of computerized data acquisition and waveform processing [7]. A recent study investigating the reliability of computerized NCS methods in a group of healthy subjects found that the reproducibility of the NCS parameters compared favorably with that from traditional electromyography laboratories [8].

The objective of the present study is to quantify the reliability of nerve conduction parameters derived entirely from computer based waveform acquisition and cursor assignments for both symptomatic and asymptomatic subjects. The goals of this study are to evaluate the overall repeatability of NCS parameters and to determine the relative contributions of test variability sources. The outcome of this study may enhance the design of future NCS instrumentation and facilitate the interpretation of NCS measurements in clinical applications.

## Methods

### Subject selection

Study subjects were recruited by advertisements at local senior centers and health clubs, by direct inquiry of volunteers participating in ongoing but unrelated clinical studies, and by word of mouth of those participating in the study. All subjects volunteered for the study and provided written informed consent. The single inclusion criterion was age 18-80 years. Women were excluded from participating if they were pregnant or nursing, as were individuals with implanted electronic medical devices. Based on responses to a symptom questionnaire administered at the start of the first testing session, a board certified neurologist (EAL) classified subjects using a four point symptom scale (0 = asymptomatic, 4 = severe symptoms). The neurologist was blinded to the electrodiagnostic data when classifying subjects. The study was approved and monitored by an independent review board (Copernicus Group, Cary, NC).

### Nerve conduction studies

The median, ulnar, deep peroneal, posterior tibial, and sural nerves were evaluated bilaterally in each subject at two test sessions 3-7 days apart. Within each session, nerves were tested twice within about 10 minutes. The same technician performed all testing. During the second test session, the technician had no access to the initial test session's results. All of the measurements were performed using a FDA approved commercial NCS/EMG instrument (ADVANCE™, NeuroMetrix, Inc., Waltham, MA). Tests were performed in a non-electrically shielded outpatient examining room with typical sources of electromagnetic interference (e.g., personal computers, 120 VAC wiring) present. Stimulating and recording electrode placement was standardized through use of integrated stimulation and recording electrodes (NeuroMetrix, Inc.) designed for use with specific nerves. The electrodes and recording methods have been described previously [9-11]. Appendix A provides a summary of the electrode configurations, stimulation and recording sites, and recording techniques.

### Nerve conduction parameters

Computerized algorithms embedded in the instrument controlled stimulus intensity, data acquisition, waveform cursor assignment, and generation of motor, F-wave and sensory nerve conduction parameters. Although the testing instrument allows full control over all aspects of stimulation, data collection, and waveform analysis, the operator was restricted from modifying data collection, waveform cursors, and measurement values. The reported parameters were stratified into primary and secondary nerve conduction parameters. Secondary parameters are nerve conduction measurements which may not be used routinely in clinical settings despite having been shown to be useful in some clinical studies. These include CMAP duration and area, F-wave chronodispersion and persistence, F-wave/CMAP amplitude ratio, and SNAP duration. While both were analyzed, only the primary parameters frequently utilized in routine clinical settings are reported herein for brevity. They include motor latencies, motor conduction velocity (CV), compound muscle action potential (CMAP) amplitude, F-wave latencies (minimum, mean, and maximum), sensory latency (DSL), sensory CV, and sensory nerve action potential (SNAP) amplitude. Motor latency is measured from stimulus onset to the initial deflection of the CMAP (i.e., negative peak onset). CMAP amplitude is measured from baseline to the negative peak. Ulnar nerve motor CV across the elbow is determined by dividing the distance between the below and above elbow cathode stimulation locations by the time difference between the corresponding below and above elbow motor latencies. The DSL is measured from stimulus onset to the initial negative peak of the SNAP. The SNAP amplitude is measured from negative to positive peak. Sensory CV was calculated only for the sural nerve.

Due to the probabilistic nature of F-wave generation, an F-wave may not be recordable following every stimulus. When a measurable F-wave is detected for the trace, F-wave onset latency is assigned. F-wave onset latency is defined as the time difference between stimulus onset and the onset of the detectable F-wave. In the upper extremity, a maximum of 10 F-waves or 12 F-wave traces were collected. In the lower extremity, a maximum of 20 F-waves were collected with a maximum of 40 (deep peroneal) and 24 (posterior tibial) traces. The F-wave mean latency is the arithmetic mean of all individual F-wave onset latencies. The F-wave maximum latency is defined as the 95^th ^percentile of the ensemble of individual F-wave onset latencies. The F-wave minimum latency is defined as the 5^th ^percentile of the ensemble of individual F-wave onset latencies. F-wave latency parameters are not reported if fewer than three F-wave onset latencies are available (five for tibial F-waves) as the parameters are not statistically robust.

Temperature compensation of raw data was performed by the instrument based on previously determined temperature correction factors [12]. Reference temperatures for compensation purposes were 32°C for median and ulnar nerves, 30°C for peroneal and tibial nerves, and 28°C for sural nerves. For example, if the skin temperature measurement from the digital thermometer embedded in the integrated electrode is 31°C for a median sensory nerve and the temperature compensation factor is -0.138 ms/°C, then a raw DSL of 4.0 ms will be compensated to 4.0 - (-0.138)*(31-32) = 3.862 ms. Repeatability analyses were performed on the data set with and without temperature compensation.

Valid results were not obtained for all parameters in every test due to technical issues such as excessive environmental noise, severe stimulus artifact, and poor signal quality. These technical issues were identified prospectively by the instrument as part of its normal operation [7] and are therefore not treated as outliers. When these technical issues were detected, the instrument did not analyze the waveforms, and nerve conduction values were not reported.

### Outliers excluded from analyses

A small number of reported measurements are identified as outliers based on a retrospective review of the data and they are grouped into three categories below. Primary analyses were performed on the temperature-compensated data set with Category I and II outliers removed. However, analyses were also carried out on the data set with all three category outliers excluded.

#### Category I outliers

Test-retest values could not be compared because the recording methodology varied between the two tests. This occurred with sural nerve testing due to two possible distances (10 cm and 14 cm) between stimulation and detection electrodes, depending on the instrument's selection of the proximal or distal pair of recording electrodes. Although the individual parameter values were valid in these cases, they could not be used to assess measurement reproducibility.

#### Category II outliers

During routine instrument operation, certain waveform cursor patterns are suggestive of unreliable waveform cursor assignments. For example, if the instrument could not assign CMAP duration cursor, the instrument operator is alerted to review all parameters associated with the CMAP waveform. In this study, parameters associated with waveforms having incomplete cursor assignments were excluded to eliminate subjectivity in operator's decision.

#### Category III outliers

During normal instrument operation, cursor assignments for waveforms are displayed in real-time. The operator is expected to review all cursor assignments and to correct any inaccurate cursor assignments to ensure accuracy of the NCS results. A retrospective analysis of all waveform cursor assignments was conducted by one of the authors (XK), and a number of these assignments were determined to be incorrect. These incorrect cursor assignments are primarily due to artifact contaminating the waveforms. The study operator was restricted from correcting any inaccurate cursor assignments in order to highlight the performance of instrument automation in this study.

### Statistical methods

The primary study outcome is the variance component analyses. A nested analysis of the variance model was used to perform a variance component analysis [13]. The two key outcome measures were within-session variance and between-session variance. The former quantifies the short-term variation in instrument measurements where all controllable sources of variation are minimized. The latter quantifies the variation between tests performed 3-7 days apart with distinct electrode placements. In this instance, sources of variability independent of the instrument were present and included differences in electrode placement and physiological changes which had occurred between the two sessions. Between-session-test variance quantifies the variation between two tests performed in two different sessions. The between-session-test variance is calculated as the sum of within-session variance and between-session variance. The square root of the variance, which has the same units as the measurements themselves, was reported for determination of the clinical relevance of these results. The within-session, between-session, and between-session-test coefficient of variation (CoV, standard deviation divided by the mean) was determined for each nerve parameter.

Additional statistical measures include the intraclass correlation coefficient (ICC) and relative intertrial variation (RIV). RIV measures the difference of repeat measurements as a percentage of their mean. For each parameter type, the 5^th ^and 95^th ^percentile of the RIV values are reported as RIV interval [14]. These statistics were calculated for pairs of tests either within a session or between two sessions. The between-session test-retest pairs were cascaded for the four possible combinations (i.e., Test 1 (T1) of Session 1 (S1) pairs with T1 of S2, T1S1 paired with T2S2, T2S1 paired with T1S2, and T2S1 paired with T2S2).

## Results

### Subject characteristics

A total of 30 subjects (15 female) participated in this study. One (female) did not complete the study and was excluded from further data analysis. The demographics of the 29 subjects are listed in Table 1. Their ages range from 22 to 77 years (mean 50.0, standard deviation (SD) 17.5 years). The mean body mass index is 25.8 (SD 5.4) with six patients obese (BMI > 30 kg/m^2^). Twenty-five subjects (86%) had symptoms, and eleven subjects (38%) had a symptom grade of 3 or greater. Twelve subjects reported occasional or frequent tingling or numbness in extremities (arm, hand, leg, and/or foot); in six of them, the symptoms extended beyond the fingers or toes to involve the hand/forearm or foot/lower leg. Eighteen subjects reported having low back pain, and nine had neuropathic pain.

**Table 1:** Demographic characteristics of subjects (n = 29)

	**Gender**	**Age (Yr)**	**Height (cm)**	**BMI (kg/m^2^)**	**Symptom Grade**	**Days between Sessions**
	
Mean	48%*	50.0	170.3	25.8	2.1	5.5
Standard Deviation	-	17.5	7.7	5.4	1.3	1.4
Minimum	-	22.0	157.5	15.9	0	3
Maximum	-	77.0	185.4	44.1	4	7

* Percentage of female subjects.

### Measurements included and excluded

Measurements of 110 nerve conduction parameters (56 primary parameters and 54 secondary parameters) were attempted for each test on a given subject. The tests were repeated once at each of the two sessions, yielding a maximum of 440 measurements per subject. Therefore, among the 29 subjects the maximum possible number of measurements was 12760. A total of 11961 measurements were reported by the instruments, leading to an overall measurement yield of 93.7%. The 799 measurements not reported were not outliers because the instrument did not provide any output values prospectively. F-wave parameters, particularly from the median and peroneal nerves, accounted for the majority of unreported measurements (540 or 67.6%). Twenty-eight (28) measurements (from 6 sural nerves) were excluded as category I outliers and 24 measurements (from 5 nerves) were excluded as category II outliers. Primary analyses were based on a data set with category I and II outliers excluded and therefore the rate of retrospective data exclusion was 0.43% (52/11961). Thirty-nine (39) measurements (from 8 nerves) were classified as category III outliers. Results mirroring the primary analyses are presented in Appendix B, computed after exclusion of all outliers (91). In this case, the rate of retrospective outlier removal was 0.76% (91/11961).

### Results of statistical analyses

Variance component analysis results are presented in Table 2 for temperature compensated NCS parameters with category I and II outliers excluded. The standard deviations of the parameters attributable to individual subject/nerve; test session; and tests within a session are calculated in addition to overall parameter standard deviation. The CoVs are tabulated for variation attributable to sessions and tests. Results for F-wave latency parameters are also listed separately for upper (median and ulnar) and lower (peroneal and tibial) extremity nerves as their mean values differ significantly. Variance component analyses were repeated for the 11 subjects with a symptom grade of 3 or greater and the results for the sub-group are presented in Table 3.

**Table 2:** Variance component analyses of nerve conduction parameters for all 29 subjects

			**Standard**	**Standard Deviation**			**Coefficient of Variation**		
					
**Parameter**	**Count**	**Mean**	**Deviation**	**Nerve**	**Session**	**Test**	**Session**	**Test**	**Session-Test**
Latency									
								
DML	910	3.49	0.82	0.79	0.24	0.12	6.8%	3.5%	7.6%
Latency Elbow	450	7.65	1.43	1.39	0.35	0.06	4.5%	0.8%	4.6%
DSL	653	3.31	0.72	0.64	0.29	0.15	8.7%	4.6%	9.8%
									
Conduction Velocity (CV)									
								
Ulnar CV Elbow	221	50.78	7.08	6.67	2.02	1.35	4.0%	2.7%	4.8%
Sural CV	200	37.12	3.80	3.45	1.59	0.75	4.3%	2.0%	4.7%
									
F-wave Latency (FWL), All Nerves									
								
Mean FWL	832	40.15	12.86	13.15	0.96	1.41	2.4%	3.5%	4.2%
Minimum FWL	801	37.92	12.17	12.63	1.16	1.53	3.1%	4.0%	5.1%
Maximum FWL	801	42.41	14.13	14.51	0.03	2.19	0.1%	5.2%	5.2%
									
Median/Ulnar Nerve FWL									
								
Mean FWL	421	28.50	3.14	2.97	0.64	0.71	2.3%	2.5%	3.4%
Minimum FWL	421	27.43	2.95	2.74	0.71	0.76	2.6%	2.8%	3.8%
Maximum FWL	421	30.29	3.63	3.27	0.82	1.34	2.7%	4.4%	5.2%
									
Peroneal/Tibial Nerve FWL									
								
Mean FWL	411	52.09	6.54	6.63	1.16	1.88	2.2%	3.6%	4.2%
Minimum FWL	380	49.54	6.75	7.00	1.50	2.10	3.0%	4.2%	5.2%
Maximum FWL	380	55.84	7.92	7.77	0.00	2.79	0.0%	5.0%	5.0%
									
Amplitude									
								
CMAP Amplitude	910	5.17	2.66	2.54	0.81	0.25	15.6%	4.8%	16.3%
CMAP Amplitude Elbow	450	5.65	1.97	1.77	0.83	0.29	14.8%	5.1%	15.6%
SNAP Amplitude	668	26.94	19.12	18.33	4.51	2.86	16.8%	10.6%	19.8%

Measurement units:

▪ Latency parameters are in milliseconds.

▪ Conduction velocities are in meters per second.

▪ CMAP amplitude is in millivolts.

▪ SNAP amplitude is in microvolts.

F-wave latency results are also presented separately for median/ulnar nerve group and peroneal/tibial nerve group as they have significantly different mean values.

Standard deviations attributable to nerves (i.e., variation among different subjects) are highest, followed by standard deviations attributable to variation between two sessions (separated by 3-7 days) for most parameters.

Standard deviations attributable to test variation are generally the lowest, with the exception of F-wave latency.

**Table 3:** Variance component analyses of nerve conduction parameters for subjects with symptom grade > 2

			**Standard**	**Standard Deviation**			**Coefficient of Variation**		
					
**Parameter**	**Count**	**Mean**	**Deviation**	**Nerve**	**Session**	**Test**	**Session**	**Test**	**Session-Test**
Latency									
								
DML	345	3.62	0.95	0.34	0.31	0.12	8.7%	3.3%	9.3%
Latency Elbow	168	7.93	1.56	0.48	0.48	0.05	6.0%	0.7%	6.0%
DSL	246	3.48	0.94	0.47	0.42	0.22	12.0%	6.2%	13.5%
									
Conduction Velocity (CV)									
								
Ulnar CV Elbow	80	48.49	7.18	2.94	2.8	0.91	5.8%	1.9%	6.1%
Sural CV	73	35.59	4.49	2.1	1.93	0.82	5.4%	2.3%	5.9%
									
F-wave Latency (FWL), All Nerves									
								
Mean FWL	311	40.58	13.17	2.14	1.37	1.64	3.4%	4.0%	5.3%
Minimum FWL	301	38.38	12.59	2.28	1.42	1.78	3.7%	4.6%	5.9%
Maximum FWL	301	42.73	14.06	2.2	0.4	2.16	0.9%	5.1%	5.1%
									
Median/Ulnar Nerve FWL									
								
Mean FWL	163	29.33	3.86	1.27	1.01	0.78	3.4%	2.7%	4.3%
Minimum FWL	163	28.15	3.51	1.34	1.12	0.73	4.0%	2.6%	4.8%
Maximum FWL	163	31.27	4.51	2.02	1.26	1.58	4.0%	5.0%	6.4%
									
Peroneal/Tibial Nerve FWL									
								
Mean FWL	148	52.97	7.37	2.79	1.62	2.28	3.1%	4.3%	5.3%
Minimum FWL	138	50.46	7.83	3.07	1.67	2.57	3.3%	5.1%	6.1%
Maximum FWL	138	56.26	8.26	2.53	0	2.53	0.0%	4.5%	4.5%
									
Amplitude									
								
CMAP Amplitude	345	5.15	2.86	0.74	0.7	0.25	13.5%	4.8%	14.3%
CMAP Amplitude Elbow	168	5.16	1.89	0.72	0.67	0.26	13.0%	5.0%	13.9%
SNAP Amplitude	255	24.61	21.73	5.85	5.42	2.22	22.0%	9.0%	23.8%

Measurement units:

▪ Latency parameters are in milliseconds.

▪ Conduction velocities are in meters per second.

▪ CMAP amplitude is measured in millivolts.

▪ SNAP amplitude is measured in microvolts.

F-wave latency results are also presented separately for median/ulnar nerve group and peroneal/tibial nerve group as they have significantly different mean values.

Standard deviations attributable to nerves (i.e., variation among different subjects) are highest, followed by standard deviations attributable to variation between two sessions (separated by 3-7 days) for most parameters.

Standard deviations attributable to test variation are generally the lowest, with the exception of F-wave latency.

Analyses were also carried out for each nerve type and the CoVs for between-session-test variation are summarized in Table 4 for commonly measured NCS parameters. The ICC and RIV are tabulated in Table 5 for tests within each session and across two sessions. The ICCs are all greater than 0.95 for within session measurements. Additional ICC results are presented by nerve type in Table 4 for NCS parameters with contribution from more than one nerve. The between-session RIV intervals of mean FWL are [-5.6%, 5.3%] for median nerves, [-7.4%, 4.6%] for ulnar nerves, [-4.6%, 10.0%] for peroneal nerves, and [-6.7%, 5.7%] for tibial nerves. The RIV intervals of SNAP amplitude are [-34.3%, 28.9%] for median nerves, [-25.5%, 25.7%] for ulnar nerves, and [-79.5%, 152.5%] for sural nerves.

**Table 4:** Repeatability performance measures by nerve type

**Parameter**	**Median**	**Ulnar**	**Peroneal**	**Tibial**	**Sural**	**Overall**
CoV for between-session-test variation						
				
DML	5.9%	4.1%	8.1%	9.5%	-	7.6%
DSL	8.0%	14.2%	-	-	4.5%	9.8%
Mean FWL	3.7%	2.9%	5.0%	3.8%	-	4.2%
Minimum FWL	4.1%	3.5%	4.6%	5.8%	-	5.1%
Maximum FWL	5.2%	5.0%	5.8%	4.0%	-	5.2%
CMAP Amplitude	7.5%	16.3%	18.7%	23.6%	-	16.3%
SNAP Amplitude	16.8%	18.3%	-	-	30.8%	19.8%
						
ICC for between-session tests						
				
DML	0.93	0.83	0.78	0.53	-	0.90
DSL	0.88	0.55	-	-	0.83	0.83
Mean FWL	0.93	0.91	0.83	0.94	-	0.98
Minimum FWL	0.91	0.87	0.85	0.85	-	0.98
Maximum FWL	0.85	0.79	0.81	0.96	-	0.98
CMAP Amplitude	0.93	0.77	0.90	0.90	-	0.91
SNAP Amplitude	0.92	0.83	-	-	0.76	0.92

**Table 5:** ICC and RIV interval for nerve conduction study parameter repeatability

	**Within Session Tests**		**Between Session Tests**	
		
	**ICC**	**RIV**	**ICC**	**RIV**
Latency				
				
DML	0.98	[-3.6%, 7.2%]	0.90	[-15.9%, 13.1%]
Latency Elbow	1.00	[-1.3%, 1.9%]	0.94	[-8.3%, 10.0%]
DSL	0.96	[-3.8%, 4.3%]	0.83	[-9.1%, 14.5%]
				
Conduction Velocity (CV)				
				
Ulnar CV Elbow	0.97	[-5.3%, 4.5%]	0.89	[-12.2%, 8.3%]
Sural CV	0.96	[-4.7%, 3.2%]	0.77	[-9.6%, 12.8%]
				
F-Wave Latency (FWL)				
				
Mean FWL	0.99	[-3.8%, 4.6%]	0.98	[-6.1%, 5.7%]
Minimum FWL	0.98	[-6.1%, 5.8%]	0.98	[-9.1%, 7.7%]
Maximum FWL	0.98	[-7.6%, 7.6%]	0.98	[-9.0%, 9.3%]
				
Amplitude				
				
CMAP Amplitude	0.99	[-9.2%, 17.7%]	0.91	[-48.4%, 37.6%]
CMAP Amplitude Elbow	0.98	[-9.0%, 10.9%]	0.83	[-34.4%, 23.4%]
SNAP Amplitude	0.98	[-11.9%, 12.5%]	0.92	[-46.0%, 31.5%]

Without temperature compensation, the within-session CoVs were about the same as that with temperature compensation for all parameters. However, the between-session CoVs increased to 9.5% for DML (compared to 6.8% with compensation), 9.8% for DSL (8.7% with compensation), and 6.6% for sural CV (4.3% with compensation). Similarly, the ICCs for between session tests decreased to 0.83 for DML (from 0.90 with compensation), 0.83 for DSL (no change), and 0.63 for sural CV (0.77 with compensation).

Thirty-nine (39) category III outliers were included in the analysis results presented in Tables 2, 3, 4 and 5. They were associated with incorrect automated cursor assignments that were readily identifiable and correctable. Excluding these outliers reduced the between-session-test CoV to 6.9% for DML, 6.3% for DSL, and 15.4% for SNAP amplitude. The ICCs for between session tests increased to 0.92 for DML, 0.93 for DSL, and 0.95 for SNAP amplitude. Complete results mirroring those in Tables 2, 3, 4 and 5 can be found in Tables 6, 7 and 8.

**Table 6:** Repeatability performance measures by nerve type with category I-III outliers removed

**Parameter**	**Median**	**Ulnar**	**Peroneal**	**Tibial**	**Sural**	**Overall**
CoV for between-session-test variations						
				
DML	5.9%	4.1%	7.6%	7.8%	-	6.9%
DSL	7.6%	4.5%	-	-	4.5%	6.3%
Mean FWL	3.7%	2.9%	5.0%	3.8%	-	4.2%
Minimum FWL	4.1%	3.5%	4.6%	5.8%	-	5.1%
Maximum FWL	5.2%	5.0%	5.8%	4.0%	-	5.2%
CMAP Amplitude	7.5%	16.3%	18.6%	23.7%	-	16.3%
SNAP Amplitude	13.4%	11.3%	-	-	30.8%	15.4%
						
ICC for between-session tests						
				
DML	0.93	0.83	0.81	0.67	-	0.92
DSL	0.89	0.90	-	-	0.83	0.93
Mean FWL	0.93	0.91	0.83	0.94	-	0.98
Minimum FWL	0.91	0.87	0.85	0.85	-	0.98
Maximum FWL	0.85	0.79	0.81	0.96	-	0.98
CMAP Amplitude	0.93	0.77	0.90	0.90	-	0.90
SNAP Amplitude	0.95	0.97	-	-	0.76	0.95

**Table 7:** Variance component analyses of nerve conduction parameters (excluding category I-III outliers)

			**Standard**	**Standard Deviation**			**Coefficient of Variation**		
					
**Parameter**	**Count**	**Mean**	**Deviation**	**Nerve**	**Session**	**Test**	**Session**	**Test**	**Session-Test**
Latency									
								
DML	904	3.50	0.82	0.80	0.22	0.11	6.2%	3.1%	6.9%
Latency Elbow	450	7.65	1.43	1.39	0.35	0.06	4.5%	0.8%	4.6%
DSL	649	3.29	0.69	0.65	0.19	0.07	5.9%	2.1%	6.3%
									
Conduction Velocity (CV)									
								
Ulnar CV Elbow	221	50.78	7.08	6.67	2.02	1.35	4.0%	2.7%	4.8%
Sural CV	200	37.12	3.80	3.45	1.59	0.75	4.3%	2.0%	4.7%
									
F-wave Latency (FWL), All Nerves									
								
Mean FWL	832	40.15	12.86	13.15	0.96	1.41	2.4%	3.5%	4.2%
Minimum FWL	801	37.92	12.17	12.63	1.16	1.53	3.1%	4.0%	5.1%
Maximum FWL	801	42.41	14.13	14.51	0.03	2.19	0.1%	5.2%	5.2%
									
Median/Ulnar Nerve FWL									
								
Mean FWL	421	28.50	3.14	2.97	0.64	0.71	2.3%	2.5%	3.4%
Minimum FWL	421	27.43	2.95	2.74	0.71	0.76	2.6%	2.8%	3.8%
Maximum FWL	421	30.29	3.63	3.27	0.82	1.34	2.7%	4.4%	5.2%
									
Peroneal/Tibial Nerve FWL									
								
Mean FWL	411	52.09	6.54	6.63	1.16	1.88	2.2%	3.6%	4.2%
Minimum FWL	380	49.54	6.75	7.00	1.50	2.10	3.0%	4.2%	5.2%
Maximum FWL	380	55.84	7.92	7.77	0.00	2.79	0.0%	5.0%	5.0%
									
Amplitude									
								
CMAP Amplitude	904	5.19	2.66	2.54	0.81	0.25	15.6%	4.8%	16.3%
CMAP Amplitude Elbow	450	5.65	1.97	1.77	0.83	0.29	14.8%	5.1%	15.6%
SNAP Amplitude	663	26.87	19.02	18.52	3.82	1.61	14.2%	6.0%	15.4%

Measurement units:

▪ Latency parameters are in milliseconds.

▪ Conduction velocities are in meters per second.

▪ CMAP amplitude is measured in millivolts.

▪ SNAP amplitude is measured in microvolts.

F-wave latency results are also presented separately for median/ulnar nerve group and peroneal/tibial nerve group as they have significantly different mean values.

Standard deviations attributable to nerves (i.e., variation among different subjects) are highest, followed by standard deviations attributable to variation between two sessions (separated by 3-7 days) for most parameters.

Standard deviations attributable to test variation are generally the lowest, with the exception of F-wave latency.

**Table 8:** ICC and RIV interval for nerve conduction study parameter repeatability with category I-III outliers removed

	**Within Session Tests**		**Between Session Tests**	
		
	**ICC**	**RIV**	**ICC**	**RIV**
Latency				
				
DML	0.98	[-3.5%, 7.2%]	0.92	[-15.3%, 12.9%]
Latency Elbow	1.00	[-1.3%, 1.9%]	0.94	[-8.3%, 10.0%]
DSL	0.99	[-3.6%, 4.1%]	0.93	[-8.6%, 14.3%]
				
Conduction Velocity (CV)				
				
Ulnar CV Elbow	0.97	[-5.3%, 4.5%]	0.89	[-12.2%, 8.3%]
Sural CV	0.96	[-4.7%, 3.2%]	0.77	[-9.6%, 12.8%]
				
F-Wave Latency (FWL)				
				
Mean FWL	0.99	[-3.8%, 4.6%]	0.98	[-6.1%, 5.7%]
Minimum FWL	0.98	[-6.1%, 5.8%]	0.98	[-9.1%, 7.7%]
Maximum FWL	0.98	[-7.6%, 7.6%]	0.98	[-9.0%, 9.3%]
				
Amplitude				
				
CMAP Amplitude	0.99	[-9.1%, 17.5%]	0.90	[-47.8%, 37.1%]
CMAP Amplitude Elbow	0.98	[-9.0%, 10.9%]	0.83	[-34.4%, 23.4%]
SNAP Amplitude	0.99	[-11.5%, 12.4%]	0.95	[-42.7%, 30.0%]

**Figure 1:**
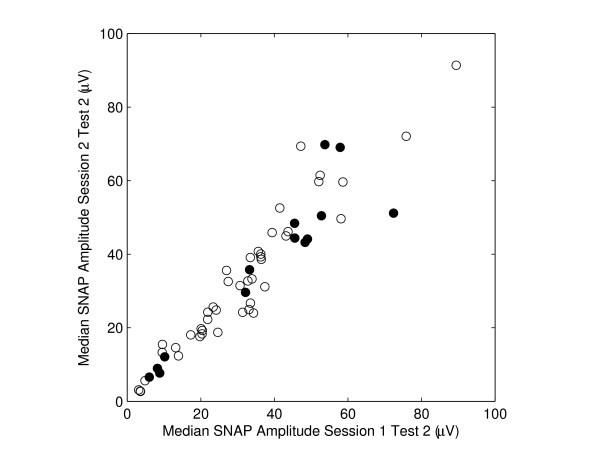
**Scatter plot of median nerve SNAP amplitude measured in two study sessions (3-7 days apart)**. Filled circles are from subjects who had complaint of frequent hand/arm pain or tingling. The intra-class correlation coefficient (ICC) is 0.95 for open circles (nerves from subjects without frequent hand/arm pain or tingling) and 0.93 for filled circles (nerves from subjects with frequent hand/arm pain or tingling).

Figure 1 presents a scatter plot of median nerve SNAP amplitude for Test 2 of the two study sessions. Filled circles are the SNAP results from subjects who had self-reported symptoms of frequent hand/arm pain or tingling. The ICC is 0.95 for nerves without stated symptoms (i.e., open circles) and 0.93 for nerves with stated symptoms (filled circles).

## Discussion

Repeat nerve conduction measurements do not generate numerically identical values because they are influenced by sources of variation. These include use of disparate equipment, differences in placement of stimulating and recording electrodes, and manual assignments of waveform cursors. The present study controlled for several of these potential sources of variation. The same instrument was used throughout the study. For tests within each session, the effect of electrode placement variation was controlled through the use of nerve specific integrated electrodes that remained in position between tests; additionally, manual editing of waveform cursors was not permitted. However, notwithstanding these controls, other sources of variation could not be mitigated and may have impacted within-session test-retest repeatability. These sources of variation included amplifier noise, Johnson noise, 60 Hz and other sources of electrical interference, short term changes in the electrode to skin interface, small unavoidable patient movements, short term physiological noise due to changes in blood flow and perspiration, and random motor neuron sampling during elicitation of F-waves. For between-session testing, the same within-session controls were in effect, with the exception that a new electrode array was applied.

Although functionality was provided by the instrument used in this study to perform real-time data acquisition control and manual editing of computer cursor assignments, this functionality was not utilized. As such, the study design eliminated the uncertainty associated with manual waveform cursor assignment. Therefore, the reliability of nerve conduction parameters in this study is exclusively based on computer based data acquisition and waveform cursor assignments. Retrospective manual review of all waveforms and computer cursor assignments identified only 39 out of 11961 cursor assignments needing corrections. As instrument operators are expected to review waveforms and cursor assignments during normal operations, repeatability performance of the instrument should be better than that reported in Tables 2, 3, 4 and 5. Indeed, results in Appendix B confirmed the performance improvement when these 39 outliers were removed from the analyses. For example, the ICC for between-session tests increased to 0.93 (from 0.83) for DSL and to 0.95 (from 0.92) for SNAP amplitude. Similarly, the between-session-test CoV was reduced by 36% (from 9.8% to 6.3%) and 22% (from 19.8% to 15.4%) respectively for DSL and SNAP amplitude.

Analysis of variance results quantify the contribution of various sources to NCS variability between measurements. The between-test CoV measures the variation in NCS measurements and therefore represents the neurophysiological variability inherent in nerve conduction testing and the variability attributable to the instrument in absence of operator intervention. In the present study, motor and sensory latencies and conduction velocities all have CoV values at or below 5%. CMAP and SNAP amplitude parameters have a higher CoV at 5-11%. Low CoV values between tests within a session (i.e., same electrode placement and retest within 10 minutes) suggest that the instrument functioned in a reproducible fashion. Between-session CoV measures are higher than between-test CoV. The between-session CoV captures the variability occurring independent of within-session variation, such as differences in electrode placement and inter-session physiological changes. Nevertheless, the CoV are still below 9% for all latency and CV parameters and around 15% for amplitude parameters. The between-session CoVs are greater than within-session CoVs for all parameters except for F-wave latencies. A higher between-test CoV for F-wave latency is a direct result of the stochastic nature of F-waves.

The between-session-test CoV quantifies the total variation between tests performed 3-7 days apart during two different sessions (with distinct electrode placements). Motor and sensory latencies, conduction velocities and F-wave latency parameters have low between-session-test CoVs ranging from 4.2% to 9.8%. These results are comparable to the reliability of similar parameters obtained in controlled multi-center clinical trials with central laboratory oversight [3,4]. The between-session-test CoV of this study is a more conservative measure of repeatability than the between-session CoV in the two benchmark studies, as the between-session-test CoV captures both between-session and between-test (within a session) variation. In this study, the motor and sensory amplitudes have between-session-test CoVs that range from 15.6 to 19.8%. These results are consistent with between-session CoV results in [3], which were 9-16%; and better than sensory amplitude between-session CoV results reported in [4], which were approximately 50%.

F-wave latency parameters exhibited similar repeatability among the studied nerves. As in other studies, the DML and CMAP amplitude parameters in this study have superior repeatability for upper extremity nerves compared to lower extremity nerves. A study by Bril and colleagues [3] showed that the CoVs for peroneal DML and CMAP amplitude were about 25% higher than those for the corresponding median nerves. A similar increase in DML is seen in this study but the CoV increase in CMAP amplitude is larger. Greater peroneal and tibial CMAP amplitude variability may be due to the small recording electrode sizes (surface areas < 2 cm^2^) used in this study [15]. For sensory responses, ulnar DSL and SNAP were disproportionably affected by the outliers and the performance levels are comparable between the median and ulnar nerves when category III outliers are excluded (see Table 6). Compared to the median nerve, the sural SNAP amplitude in this study exhibited lower reliability (ICC of 0.92 for median versus 0.76 for sural), similar to data (ICC of 0.83 for median versus 0.77 for sural) reported elsewhere [14].

When ranked by the CoV, F-wave latencies have the best reproducibility (≤ 5%), followed by conduction velocity and latency parameters (<10%). Amplitudes have lower reproducibility; however, the CoVs are still below 20%. This repeatability hierarchy is consistent with the multicenter study by Kohara and colleagues in which they concluded that the minimum F-wave latency was the most reproducible nerve conduction measure for assessing diabetic neuropathy using manual electrodiagnostic methods [14]. A study by Bird and colleagues [4] showed a similar pattern of repeatability ranking for various NCS parameters using data from a multi-center trial: The CoV was lowest for F-wave latency (8-11%), followed by motor and sensory conduction velocity (8-12%), and finally sensory amplitude (52-53%).

Uniformly high intraclass correlation coefficients (ICC) are observed for all NCS parameters when two tests within a session are compared. When tests from different sessions are compared, the ICC is highest for F-wave latencies (0.98), and slightly lower for motor and sensory latencies (0.83-0.94), amplitudes (0.83-0.92), and conduction velocities (0.77-0.89). These results are comparable to the results reported in other repeatability studies [4,14,16]. For example, the ICC for median nerve minimum F-wave latency is 0.91 in this study while an ICC of 0.93-0.94 was reported for the minimum F-wave latency in the study by Kohara et al. [14] and 0.87-0.90 in the study of Bird et al. [4]. In this study, the lowest amplitude ICC is 0.76 for the sural nerve. Similar sural amplitude ICC values were reported as 0.76-0.80 in [4], 0.77 in [14], and 0.74 in [16].

RIV intervals were reported as a repeatability metric in two other studies [14,16]. F-wave latency parameters have the narrowest RIV intervals among all NCS parameters in the present study, similar to the results from the aforementioned studies. The RIV intervals of mean F-wave latencies are narrower than those in the study of Pinheiro et al. for the ulnar and tibial nerves [16]. CMAP and SNAP amplitudes have the widest RIV intervals in the present study, matching the results reported in the other two studies. For example, the RIV interval for tibial CMAP amplitude is [-68.5%, 46.2%], compared with [-89%, 75%] in the Pinheiro study, and [-35%, 40%] in [14].

Skin temperature changes have a predictable effect on motor and sensory latencies [17]; however, control of skin temperature is often difficult to achieve. In this study, rather than warming or cooling extremities to achieve uniform skin temperature, the impact of skin temperature was mitigated by mathematical compensation within a predetermined range. Temperature compensation does not have a material impact on test-retest repeatability within a session as measured by between-test CoV since any temperature variation between the two tests 10-minute apart are negligible. However, temperature compensation improves reproducibility for tests between two sessions 3-7 days apart, as the temperature variation can be significant. The CoVs of the DML and DSL decrease by 28.4% and 11.2% respectively, with temperature compensation. The sural conduction velocity CoV decreases by 34.8%, and mean F-wave latency CoVs decreases by 14.3%. Similar improvements are noted using ICC and RIV intervals.

A total of 799 of 12760 (6.3%) NCS measurements were prospectively rejected by the electrodiagnostic instrument. These rejected measurements are not outliers as they were not processed and reported by the instrument. The reasons for these rejections were poor signal quality or artifact interfering with the evoked response. This rate of rejection is comparable to the rate observed in controlled studies of nerve conduction reproducibility with central laboratory oversight. Bril and colleagues reported an average trace rejection rate of 6.3 to 8.7% [3]. In another study with similar design, Bird and colleagues reported a trace rejection rate of 4% [4].

During a retrospective review of the data, 52 measurements were flagged as unsuitable for repeatability performance evaluation (category I and II outliers defined in Method section). An additional 39 measurements were identified as outliers due to inaccurate cursor assignments (category III outliers). Because this study was designed to assess the reproducibility of computer-generated nerve conduction parameters without human intervention (although such intervention is possible with the instrument used), the inaccurate cursor assignments affecting 39 NCS parameters were left unchanged rather than manually corrected. The rate of outliers in this study (39/11961, 0.33%) is low compared to the rate of trace corrections reported in the benchmark studies; 34.3 - 38.2% in [3]; and 19% in [4]. In practice, outliers are expected to be corrected manually during operator review of NCS waveforms, and these corrections should lead to an improvement in repeatability performance metrics.

## Conclusion

In this study the reproducibility of nerve conduction parameters is assessed quantitatively based on data acquired by an electrodiagnostic instrument using computer based waveform cursor assignment. The results are benchmarked against those from nerve conduction reproducibility studies in which the nerve conduction measurements were performed manually with central laboratory review [3,4,14,16]. The reliability of motor and sensory latencies, conduction velocities, F-wave latencies, and motor and sensory amplitudes are comparable to the results obtained in the benchmark studies. Furthermore, the ranking of reliability, whereby F-wave latencies have the best reproducibility and amplitudes the worst, is also consistent with the benchmark studies. The study demonstrates that nerve conduction parameters acquired with modern electrodiagnostic instruments are highly repeatable. The repeatability of nerve conduction measurements make them ideal candidates for longitudinal tracking of peripheral nerve function, such as in monitoring neuropathic disease progression, assessing the efficacy of interventions in clinical trials, and detecting peripheral neurotoxicity.

## Competing interests

The authors are employees of Neurometrix, Inc.

## Authors' contributions

All authors have contributed to and approved the final manuscript. SNG and XK conceived the designed the study. XK and EAL developed the methodology and acquired the data. XK conducted statistical analysis and wrote the first draft with SNG.

## Appendix A. Description of Nerve Specific Electrodes and Recording Techniques

The electrodes used in this study are commercially available, pre-fabricated, nerve specific, surface electrode arrays (NeuroMetrix, Inc., Waltham, MA). In addition to stimulating (cathode and anode) and recording (active, inactive, and reference) electrodes, each nerve specific electrode array (NSE) incorporates a digital thermometer, a unique digital identifier, printed electrical traces, and a keyed connector.

### Median NSE

When a median NSE is properly affixed to the patient's skin utilizing anatomic landmarks, the stimulation cathode is located over the median nerve at the midline volar wrist, 3.0 centimeter (cm) proximal to the distal wrist crease. The 2.4 cm by 2.4 cm active recording electrode is located over the motor point of the abductor pollicis brevis, while the inactive recording electrode is positioned over the interphalangeal joint of the thumb. Concurrent with acquisition of motor and F-wave responses, an antidromic sensory response is recorded from the middle finger (digit 3) using ring electrodes. The active electrode is located over the proximal interphalangeal (PIP) joint, with the inactive electrode 3.0 cm more distal. Inter-electrode distances are measured from center to center.

### Ulnar NSE

When an ulnar NSE is properly affixed to the patient's skin utilizing anatomic landmarks, the stimulation cathode is located over the ulnar nerve at the medial volar wrist, 3.0 cm proximal to the distal wrist crease. The 1.0 cm by 2.0 cm active recording electrode is located over the motor point of the abuductor digiti minimi, while the inactive recording electrode is positioned over the lateral volar wrist. Concurrent with acquisition of motor and F-wave responses, an antidromic sensory response is recorded from the small finger (digit 5) using ring electrodes. The active electrode is located over the proximal interphalangeal (PIP) joint, with the inactive electrode 2 cm more distal. Proximal ulnar motor responses (CMAPs) are elicited by stimulating the nerve above and below the elbow. The distance between the above and below elbow stimulation cathodes is fixed at 10.5 cm [18], and stimulation is applied at the below elbow cathode prior to the above elbow cathode. An arm support fixture is used during proximal ulnar nerve stimulation to ensure arm flexion of 110 degrees measured between the upper arm and forearm [18,19]. An electronic adapter links the proximal stimulation sites to the distal stimulation and recording integrated electrode.

### Peroneal NSE

When a peroneal NSE is properly affixed to the patient's skin utilizing anatomic landmarks, the stimulation cathode is located over the deep peroneal nerve where the lateral margin of the tibia intersects the intermalleolar line. The CMAP and F-waves are recorded using two pairs of interspersed recording electrodes placed along a line between the lateral malleolus and the third toe, over the extensor digitorum brevis muscle. Two pairs of electrodes are utilized to enable a four electrode motor response scanning capability. The four electrodes are of equal size (0.7 cm by 2.7 cm). The distance between the two electrodes of each pair is 2.5 cm. Motor responses from both detector pairs are compared to determine which active electrode is directly over the motor point [20]. Subsequent acquisitions of motor response parameters are made from this electrode, with the other electrode of the pair acting as an "inactive" electrode.

### Tibial NSE

When a tibial NSE is properly affixed utilizing to the patient's skin using anatomic landmarks, the stimulation cathode is located over the posterior tibial nerve just posterior to the medial malleolus. The motor response is recorded using a detector pair located just distal to the medial malleolus with electrode size of 0.7 cm by 2.2 cm. The active electrode is located anterior to the inactive electrode. This detector configuration records the intermediate/far-field potential from non-moving dipole sources in tibial innervated foot muscles [21,22]. The resulting motor response is similar in shape and magnitude to those recorded directly over abductor hallucis--the muscle from which the tibial motor response is typically recorded from [20].

### Sural NSE

When a sural NSE is properly affixed to the patient's skin utilizing anatomic landmarks, the stimulation cathode is positioned over the sural nerve in the midline lower calf, while the *proximal active electrode *is positioned posterior to the lateral malleolus. A *proximal inactive electrode *is located 1.5 cm inferior to the active electrode (referencing standard anatomic position), measured center-to-center. A second pair of recording electrodes is positioned inferior to the lateral malleolus, also with 1.5 cm inter-electrode separation. All four electrodes are 1.0 cm by 3.5 cm. The two active electrodes are 4 cm apart. The device analyzes the antidromic sensory response recorded from the proximal pair unless the waveform quality is poor, in which case responses recorded from the distal pair are analyzed.

## Appendix B. NCS Repeatability Performance with Exclusion of Outliers

During the normal course of operation, the NCS instrument displays waveforms and cursor assignments in real-time immediately after the waveforms are acquired and analyzed. The instrument operator is expected to review the waveforms and manually correct any inaccurate cursor assignments. The study design eliminated the manual correction step in order to obtain the reliability of nerve conduction parameters derived from data acquired and analyzed exclusively by a computer. Thirty-nine (39) out of 11961 cursor assignments were found to be in need of correction based upon a manual review. With the 39 outliers removed, we report the analysis results mirroring those presented in main text in order to enumerate the true repeatability performance metrics of the NCS instrument when manual waveform reviews are allowed.

Repeatability analysis results are presented in Tables 6, 7 and 8 for temperature compensated NCS parameters with category I-III outliers excluded. Table 6 summarizes the between-session-test CoVs for commonly measured NCS parameters of each nerve type, together with ICC for the same parameters. Variance component analysis results are presented in Table 7. Motor and sensory latencies and conduction velocities all have between-test CoV values at or below 5%. CMAP and SNAP amplitude parameters have a slightly higher CoV at 5-6%. Motor and sensory latencies, conduction velocities and F-wave latency parameters have low between-session-test CoVs ranging from 4.2% to 6.9%. The SNAP amplitude between-session-test CoV is reduced to 15.4% from 19.8% when 5 readily identifiable category III outliers (out of 668 tests) are excluded. The ICC and RIV are tabulated in Table 8 for tests within each session and across two sessions. The ICCs are all greater than 0.95 for within session measurements. For between session measurements, the ICCs are no less than 0.92 for latencies, 0.83 for amplitudes, and 0.77 for CV.
